# Is Intralesional Methotrexate an Effective Alternative to Intralesional Triamcinolone in Alopecia Areata? Findings From a Randomized Controlled Trial

**DOI:** 10.1111/jocd.70367

**Published:** 2025-07-30

**Authors:** Narges Ghandi, Atiyeh Rashidi, Fatemeh Saberi, Robabeh Abedini, Nasim Tootoonchi, Hanie Babaie

**Affiliations:** ^1^ Autoimmune Bullous Diseases Research Center, Razi Hospital Tehran University of Medical Sciences Tehran Iran; ^2^ Department of Dermatology, Razi Hospital Tehran University of Medical Sciences Tehran Iran; ^3^ Center for Research and Training in Skin Diseases and Leprosy Tehran University of Medical Sciences Tehran Iran

**Keywords:** androgenetic alopecia, corticosteroids, intradermal injection, treatment

## Abstract

**Background:**

Alopecia areata (AA) is an autoimmune condition resulting in hair loss, sometimes just in small patches but occasionally across larger areas like the entire scalp. For localized AA, treatments often involve injecting corticosteroids, such as triamcinolone acetonide (TrA), directly into the affected areas. Methotrexate (MTX), a drug known for its ability to suppress immune responses, has also been considered as an alternative. However, there has not been much research directly comparing these two treatments.

**Methods:**

This study involved 40 individuals with localized AA. These patients were divided into two groups: one received TrA injections, and the other was given MTX. Both groups were treated once a month for 3 months. We tracked their progress using the Severity of Alopecia Tool (SALT) over 6 months. Their trichoscopic findings, adverse effects, and satisfaction with treatment were also documented.

**Results:**

Cases receiving TrA showed strong improvements, with their SALT scores dropping by an average of 54.36%, which meant significant hair regrowth. In contrast, the MTX group saw their scores worsen by 54.6%, meaning losing more hair. For trichoscopic changes, both groups showed some progress, but only the reduction in black dots in the MTX group was statistically significant. Side effects like mild redness or pigmentation changes were uncommon and similar for both groups. When asked how satisfied they were, patients who received TrA gave a much higher score:7.1 out of 10 compared to 4.9 for MTX.

**Discussion:**

According to our results, TrA outperformed MTX in treating localized AA, both in terms of hair regrowth and patient satisfaction. While MTX may have potential as a therapy, its inconsistent performance in this study suggests it is not ready to be a primary option for localized AA. Future research is needed to explore whether adjusting doses or combining it with other treatments could result in better outcomes.

## 1 Introduction

Alopecia areata (AA) is an inflammatory, chronic, immune‐mediated condition that causes hair loss without leaving scars [1]. The disease progression is unpredictable; however, cases with limited, short‐term hair loss have a higher chance of spontaneous remission within 1 year, and 14%–25% of individuals may get worse and lose all of their scalp hair (alopecia totalis/AT) or body hair (alopecia universalis/AU) [2].

AA appears to be the result of complicated interactions between immune system dysregulation, genetic susceptibility, and environmental triggers. The human leukocyte antigen (HLA) class I and II genes are identified as significant contributors to the development of AA [3]. Typically, hair follicles express low levels of major histocompatibility complex (MHC) molecules, which shield them from immune system attacks. In AA, this immunological privilege is compromised by inflammation, and that triggers the activation of CD8^+^ T cells; these T cells then exacerbate the damage by releasing cytokines like IL‐15. Antigen‐presenting cells then process and present autoantigens derived from the hair follicles; the presence of these autoantigens causes the immune system to lose its tolerance, ultimately triggering an autoimmune response that disrupts the hair growth cycle [1, 4].

Various treatment options have been introduced for AA over the years; recently, baricitinib and ritlecitinib got FDA‐approved for treating AA [1]. Although these two medications received FDA approval, they can cause serious adverse effects such as increased severe infections and malignancies [5, 6] and are not very budget‐friendly options. As a result, they are not good choices for patients with limited patchy AA, which emphasizes the need to look for appropriate alternatives.

Corticosteroids are one of the most used treatments for AA. They can be administered in various forms, including oral, topical, intramuscular, intravenous, and intralesional methods depending on the severity of the disease and the patient's best interest [7]. Intralesional corticosteroid (ILCS), particularly triamcinolone acetonide, is the preferred first‐line treatment for adult patients with less than 50% scalp hair loss [8]. Direct delivery to the involved area and a lower risk of systemic side effects are advantages of this administration technique [2].

Intralesional triamcinolone acetonide (TrA), administered at concentrations between 5 and 10 mg/mL every 4–6 weeks, has shown effectiveness in about 60% to 67% of patients. For areas like the eyebrows and beard, lower concentrations of 2.5–5 mg/mL are often used. New hair growth typically appears within 6–8 weeks. Injections are repeated until full hair regrowth is achieved; however, discontinuation of therapy is advised if no improvement is seen within 6 months. Interestingly, a study comparing different concentrations, (2.5 mg/mL, 5 mg/mL, and 10 mg/mL) found that the rate of hair regrowth was similar, regardless of the concentration used [9]. The most common adverse effects noted during ILCS therapy are pain, atrophy of skin, telangiectasia, cushingoid feature, and hypopigmentation [8].

Methotrexate (MTX) is one of the commonly prescribed immunosuppressive medications by dermatologists that can be administered either subcutaneously or orally. The most common side effects are gastrointestinal symptoms such as nausea, vomiting, mucosal ulcers, and appetite loss [10]. The subcutaneous injection has shown greater advantages than oral consumption, including reduced gastrointestinal complications, decreased risk of accidental oral overdose, improved tolerability, and increased and longer drug availability [11]. Oral MTX has been used to treat AA either alone or in conjunction with other therapies for years [1, 12, 13, 14, 15, 16]; however, there are only a few studies evaluating the efficacy and safety of intralesional MTX [17, 18, 19].

Comparing the effectiveness of intralesional MTX and TrA in treating AA offers valuable insights for dermatologists when treating focal AA. Intralesional corticosteroids are generally accepted as first‐line treatment but may not be an option for all patients because of contraindications or side effects. Investigating alternative therapies such as MTX may help offer a steroid‐sparing treatment for steroid‐intolerant or nonresponsive patients.

Given the advantages of direct intralesional injection and the absence of related randomized clinical trials (RCTs) evaluating the long‐term efficacy and side effects of intralesional MTX and TrA, two of the most commonly used treatments for AA, this trial is intended to provide clinically relevant data for evidence‐based treatment decisions in standard clinical practice.

## 2 Method and Material

### 2.1 Participants

The study population consisted of participants with a confirmed clinical diagnosis of AA. The Cochran formula was used to estimate and confirm the sample size. Considering an alpha of 5% and a beta of 20%, 40 people were included in the study. The sample size was estimated based on similar studies, with 20 people per group.

This study included patients aged 16–60 years with patchy AA diagnosed by a dermatologist based on clinical and trichoscopic findings, involving less than 20% of the scalp, and who had not received any treatment for AA in the past month. Pregnant and breastfeeding women, and patients with chronic diseases such as liver or blood disorders, immune deficiencies, or infectious diseases were excluded.

### 2.2 Study Design and Procedures

All required information including demographic data (age, gender, and family history), findings associated with AA and other essential information, such as disease duration, nail involvement, complications, and severity of the disease was collected in a checklist. Clinical and trichoscopic examinations were performed by a dermatologist to diagnose the location, size, and number of AA lesions, and these findings were also recorded in the checklist. Severity of AA was evaluated by a specialist using the Severity of Alopecia Tool (SALT) score as the main outcome measure. The SALT score is calculated by evaluating the percentage of hair loss in different areas of the scalp, including the top, parietal, and occipital regions. In this study, it was assessed as the mean percentage change (Primary SALT—Secondary SALT/Primary SALT * 100).

Patients were randomized to the intralesional MTX or TrA group using a computer‐generated simple randomization list. The patients and the investigators were blinded to treatment groups. Because of the variation in color and viscosity of the injected drugs, the physician who was treating was not blinded but was not involved in outcome measurements. All assessments, including SALT score and trichoscopy, were made by another blinded investigator.

In the MTX group, a vial of MTX containing 25 mg/mL was used. MTX was injected intradermally into the lesion and one centimeter around it, with an injection volume of 0.02 mL per site. A maximum dose of 0.1–0.2 mL (2.5–5 mg) was administered per session using a 0.5‐inch, 30‐gauge needle. In the corticosteroid group, the TrA (40 mg/mL) diluted to one‐fifth (8 mg/mL) and injected intradermally into the patches and one centimeter around it, with an injection volume of 0.05–0.1 mL per site. A maximum dose of 2 mL (10 mg) was administered per session using a 0.5‐inch, 30‐gauge needle. Both groups received treatments in three monthly sessions and were observed for adverse effects and treatment progress. Follow‐up visits included physical examination, trichoscopic evaluation, and adverse event reporting.

The results of the trichoscopic assessment and SALT scores (mean percentage changes) at 6 months post‐treatment were considered as the main treatment outcomes and compared with each other and baseline findings. Potential adverse effects of the drugs were also assessed, and patient satisfaction with the treatment was evaluated using a 10‐point Likert scale.

Routine laboratory evaluations, including complete blood count (CBC), liver function tests (LFTs), and kidney function assessments, were conducted for all participants at study initiation. The purified protein derivative (PPD) test and viral markers were also assessed in the MTX group. Blood MTX levels were monitored at least once following the initial injection. Subsequent CBC and LFTs were repeated at one and three months post‐treatment for those undergoing MTX therapy.

### 2.3 Ethical Considerations

After providing complete explanations about the study procedures, benefits, and potential risks based on the latest version of the Helsinki Declaration, written consent was obtained from all participants. Additionally, all study procedures were conducted in accordance with the latest version of the Helsinki Declaration on ethical principles for medical research involving human subjects. This study is authorized by the Ethics Committee of the Tehran University of Medical Sciences and Razi Hospital Research. The ethical code is IR. TUMS.MEDICINE.REC.1400.1311 and the trial code is IRCT20141209020250NG.

### 2.4 Data Analysis

After data collection, the study findings were analyzed using SPSS software version 23. For describing quantitative findings, central tendency indices such as mean and standard deviation were used, and for describing categorical data, frequency and percentage were utilized. For analytical purposes, independent t‐test and chi‐square test were employed. A *p*‐value of less than 0.05 was considered the level of significance in all stages of the study.

## 3 Results

In this clinical trial study, 40 cases of AA were divided into two treatment groups, with 20 participants in each group. The first group was treated with intralesional TrA, while the second group received intralesional MTX. Of all patients, 31 (77.5%) were male and the remaining 9 (22.5%) were female, with an average (SD) age of 34.9 (10.5) years. The average (SD) age and the mean (SD) duration of disease for the 20 patients in the TrA group were 34 (10.5) years and 2.4 (1.2) years, respectively. Of these patients, 14 (70%) were male and 6 (30%) were female. The 20 patients in the MTX group consisted of 17 (85%) males and 3 (15%) females; the average (SD) age of them was 35.9 (10.6) years, and their mean (SD) duration of disease was 2.7 (2.2) years. There were no significant differences between the two groups in terms of age, gender, family history of AA, duration of disease, nail involvement, or history of other comorbidities. Therefore, the two groups can be considered comparable (Table 1).

**TABLE 1:** Demographic characteristics of patients in each group.

	All participants (40 patients)	TrA group (20 patients)	MTX group (20 patients)	*p*
Age, year, median (SD)	34.9 (10.5)	34 (10.5)	35.9 (10.6)	0.563
Gender, *n* (%)				
Male	31 (77.5%)	14 (70%)	17 (85%)	0.451
Female	9 (22.5%)	6 (30%)	3 (15%)	
FH of AA, *n* (%)	3 (7.5%)	2 (10%)	1 (5%)	1.00
Underlying disorder, *n* (%)	1 (2.5%)	1 (5%)	0	1.00
Duration of disease^a^, year, median (SD)	2.5 (1.8)	2.4 (1.2)	2.7 (2.2)	0.599
Nail involvement^b^, *n* (%)	1 (2.5%)	1 (5%)	0	1.00

*Note: p*‐value < 0.05: Significant.

Abbreviations: FH, family history; MTX, methotrexate; SD, standard deviation; TrA, triamcinolone acetonide.

^a^ Independent *t*‐test.

^b^ Chi‐squared test.

Among patients receiving TrA, the mean SALT score was 12 (SD = 3.34) before treatment, with a median of 10.5 and an interquartile range (IQR) of 5. After 6 months, these values decreased to a mean of 5.75 (SD = 4.37), a median of 5, and an IQR of 0. In contrast, patients in the MTX group had a mean SALT score of 14.6 (SD = 3.56) before treatment, with a median of 15 and an IQR of 7.75. After 6 months, the mean score increased to 21.00 (SD = 8.2), with a median of 20 and an IQR of 10. The change in SALT scores was significant in both the TrA group (*p* = 0.001) and the MTX group (0.006). The mean SALT score percentage change turned out to be 54.36 (SD 28.7) in TrA group and −54.60 (SD 77.9) in the MTX group (*p* = 0.001). These findings suggest that patients treated with intralesional TrA experienced an average of 54.36% reduction in their SALT scores, indicating the effectiveness of TrA for decreasing hair loss among patients. The standard deviation of 28.7 reflects moderate variability in responses within this group. Conversely, patients treated with intralesional MTX showed an average increase of 54.60% in their SALT scores. This negative change signifies that, on average, hair loss worsened over time. The large standard deviation of 77.9 reveals considerable variability in treatment responses within the MTX group (Table 2).

**TABLE 2:** Comparison of SALT score and SALT% changes between two groups before and after treatment.

	First SALT score^b^, mean (SD)	Last SALT score^c^, mean (SD)	SALT % change^d^ mean (SD)	*p* ^a^
TrA group	12 (3.34)	5.75 (4.37)	54.36 (28.7)	0.001
MTX group	14.6 (3.56)	21.00 (8.2)	−54.60 (77.9)	0.006
*p*‐value^a^	0.022	0.001	0.001	

*Note: p*‐value < 0.05: significant.

Abbreviations: MTX, methotrexate; SD, standard deviation; TrA, triamcinolone acetonide.

^a^ Independent *t*‐test.

^b^ SALT score before treatment.

^c^ SALT score 3 months after last session.

^d^ [(First SALT score—last SALT score)/first SALT score] *100.

Trichoscopic findings including yellow dots, black dots, white dots, exclamation marks, empty follicles, absent follicular opening, and short vellus hair before treatment, after 3 months, and after 6 months are summarized in Table 3. Trichoscopic findings had a downward trend in both groups, except for the persistence of white dots in the MTX group and exclamation mark hairs in the TrA group. Only the reduction in black dots in the MTX group was statistically significant, and while the MTX group showed a greater overall decrease in trichoscopic findings, the difference compared to the TrA group was not statistically significant.

**TABLE 3:** Trichoscopic changes of each group before and after treatment.

Trichoscopic features	Treatment group	Before treatment	After 3 months	After 6 months	*p* ^a^
Yellow dots	TrA	14 (70%)	12 (60%)	12 (60%)	0.728
	MTX	13 (65%)	11 (55%)	8 (20%)	0.113
	*p*‐value	0.731	0.605	0.206	
Black dots	TrA	8 (40%)	4 (20%)	7 (35%)	0.740
	MTX	12 (60%)	12 (60%)	5 (20%)	0.025
	*p*‐value	0.264	0.022	0.731	
White dots	TrA	6 (30%)	4 (20%)	5 (25%)	0.721
	MTX	4 (20%)	5 (25%)	4 (20%)	1.00
	*p*‐value	0.048	1.00	1.00	
Exclamation mark hairs	TrA	5 (25%)	5 (25%)	5 (25%)	1.00
	MTX	8 (40%)	6 (30%)	2 (10%)	0.065
	*p*‐value	0.311	0.648	0.407	
Empty follicles	TrA	14 (70%)	14 (70%)	10 (50%)	0.179
	MTX	12 (80%)	11 (73.3%)	9 (45%)	0.427
	*p*‐value	1.00	1.00	0.667	
Absent follicular opening	TrA	1 (5%)	1 (5%)	0 (0%)	1.00
	MTX	1 (5%)	0 (0%)	0 (0%)	1.00
	*p*‐value	1.00	1.00		
Short vellus hairs	TrA	17 (85%)	15 (75%)	13 (65%)	0.273
	MTX	18 (90%)	19 (95%)	17 (85%)	1.00
	*p*‐value	1.00	0.182	0.273	

*Note: p*‐value < 0.05: significant.

Abbreviations: MTX, methotrexate; TrA, triamcinolone acetonide.

^a^ Chi‐squared test.

The only adverse effects were erythema (reported by 3 (15%) of cases in each group) and hypopigmentation (observed in only 1 (5%) patient in the MTX group). There was no significant difference between the two groups regarding adverse effects (Table 4).

**TABLE 4:** Comparison of adverse events and treatment satisfaction between patients receiving MTX and TrA.

Adverse effects, *n* (%)	TrA group	MTX group
Erythema	3 (15%)	3 (15%)
Hypopigmentation	0 (0%)	1 (5%)
None	17 (85%)	16 (80%)
*p* value = 0.597		
Treatment satisfaction score (from 1 to 10), mean (SD)	7.1 (2.2)	4.9 (1.9)
*p* value = 0.001		

*Note: p*‐value > 0.05: insignificant.

Abbreviations: MTX, methotrexate; TrA, triamcinolone acetonide.

The assessment of patients' satisfaction with treatment showed a significantly higher satisfaction with TrA than MTX. The TrA group's mean (SD) satisfaction score was 7.1 (2.2), whereas the MTX group's mean was 4.9 (1.9) (*p* < 0.05).

No significant associations were found between gender, age, or disease duration and the mean percentage change in SALT score, treatment satisfaction, or adverse effects within each treatment group (Table 5).

**TABLE 5:** Assessment of any associations between sex, age, disease duration with mean SALT score percentage changes, treatment satisfaction, and adverse effects in each treatment group.

	TrA									MTX								
	Sex			Age			Disease duration			Sex			Age			Disease duration		
Mean (SD) SALT score percentage changes^a^	M (14 Pt)	F (6 Pt)		> 35 years (12 Pt)	< 35 years (8 Pt)		> 2.5 years (9 Pt)	< 2.5 years (11 Pt)		M (17 Pt)	F (3Pt)		> 35 (10 Pt)	< 35 (10 Pt)		> 2.5 years (8 Pt)	< 2.5 years (12 Pt)	
	55.6% (23.2)	51.4% (41.6)		59.4% (24.2)	46.9% (34.8)		49.4% (23.2)	58.4% (41.6)		−35% (55.5)	−165% (105.7)		−45.6% (82.4)	−63.6% (76.4)		−58.9% (86.9)	−51.7% (75)	
*p* value	0.770			0.345			0.506			0.160			0.619			0.844		
Treatment satisfaction score, mean (SD)	M	F		> 35	< 35		> 2.5 years	< 2.5 years		M	F		> 35	< 35		> 2.5 years	< 2.5 years	
	7.4 (2.2)	6.4 (1.8)		7.6 (2.4)	6.3 (2)		6.8 (2.9)	7.3 (3.1)		5.1 (2.1)	3.8 (1.9)		5.9 (2.6)	3.9 (2)		4.2 (2)	5.4 (2.2)	
*p* value	0.341			0.222			0.716			0.369			0.070			0.232		
Existence of adverse effects, *n* (%)		M	F		> 35	< 35		> 2.5 years	< 2.5 years		M	F		> 35	< 35		> 2.5 years	< 2.5 years
	Yes	2 (14.3%)	1 (16.6%)	Yes	2 (16.6%)	1 (12.5%)	Yes	1 (11.1%)	8 (88.9%)	Yes	4 (23.5%)	0 (0%)	Yes	2 (20%)	2 (20%)	Yes	2 (25%)	2 (16.6%)
	No	12 (85.4%)	5 (83.3%)	No	10 (83.3%)	7 (87.5%)	No	2 (18.1%)	9 (81.9%)	No	13 (76.4%)	3 (100%)	No	8 (80%)	8 (80%)	No	6 (75%)	10 (83.3%)
*p* value	0.891			1.00			0.660			0.643			0.513			0.450		

*Note: p*‐value > 0.05: insignificant.

Abbreviations: F, female; M, male; MTX, methotrexate; Pt, patients; SALT, severity of alopecia tool; SD, standard deviation; TrA, triamcinolone acetonide.

^a^ [(First SALT score – last SALT score)/first SALT score] *100.

## 4 Discussion

AA is believed to be an autoimmune disorder that targets hair follicles, resulting in hair loss. It can affect both genders and people of any age, affecting hair on the head and other regions of the body with a lifetime chance of approximately 2% [9]. One of the biggest issues with this disorder is sudden relapses; chances are 52% for patients with childhood‐onset, 44% for adult‐ onset, and 30% for late‐onset cases, respectively [20].

Different treatments are available, ranging from traditional therapies including topical, intralesional, and oral corticosteroids, contact immunotherapy like diphenylcyclopropenone (DPCP) and squaric acid dibutyl ester (SADBE), systemic immunosuppressants such as MTX and cyclosporine, to emerging, innovative approaches like Janus kinase (JAK) inhibitors (e.g., upadacitinib, ritlecitinib, tofacitinib, baricitinib), THelper 2 pathway inhibitors (dupilumab), prostaglandin analogs (latanoprost and bimatoprost), and platelet‐rich plasma (PRP) injections, but finding an effective treatment for alopecia is an ongoing challenge [1].

Intralesional TrA is one of the most popular treatments, especially for treating limited patches of AA, but the research has not consistently shown its effectiveness [21].

Another widely used medication in dermatology is MTX, an immunosuppressant that effectively blocks the action of dihydrofolate reductase, inhibiting the synthesis of DNA and RNA, which then prevents cell survival and proliferation. It also has anti‐inflammatory properties, possibly due to increasing intracellular and extracellular amounts of adenosine [22] and decreasing lesional TNF‐a [23] While intralesional injection of MTX seems like a good administration technique for delivering the medication directly to the affected area and reducing systematic adverse effects, there are a handful of trials assessing the effectiveness and side effects [17, 18, 24].

We conducted this RCT in order to evaluate and compare the long‐term effectiveness and side effects of intralesional TrA and MTX. Two patient groups (each group consisted of 20 AA cases) with comparable clinical and demographic characteristics participated in our investigation. Patients received 3 sessions of treatment, 4 weeks apart; their SALT score was evaluated before treatment and 6 months after.

Our results underscore a clear distinction in the efficacy of TrA versus MTX. Patients treated with TrA showed a significant reduction in their SALT score percentage, with an average decrease of 54.36% (SD 28.7), while patients in the MTX group experienced an average (SD) increase of 54.6% (77.9) in SALT score percentage. *p* = 0.001 indicates this difference is statistically significant.

Although TrA seems highly effective in stimulating hair regrowth in AA patients, MTX does not show promising outcomes. Additionally, the large standard deviation in the MTX group's SALT score percentage change (77.9) reflects considerable variability in patient responses.

Hamdino et al. [17] compared these two treatments for the first time. According to their results, 20% of patients in the MTX group and 40% in the TrA group (there were 20 cases in each group) saw more than 75% hair regrowth after 3 months of therapy (*p*‐value = 0.028), and at the 3‐month follow‐up treatment, 65% of patients in the MTX group and 50% in the TrA group experienced more than 75% hair regrowth (*p* = 0.153). They declared that intralesional MTX may be as effective as intralesional TrA in treating people with localized AA.

Another recent trial carried out by Rafique et al. [24] involved the intralesional administration of TrA to 64 patients and MTX to 64 patients. There was no evident difference (*p* = 0.155) in the number of patients who achieved remission after 3 months between the MTX group's 56 (87.5%) and TrA group's 61 (95.3%). Although it was not statistically significant, the recurrence percentage was greater in the MTX group (10.9% versus 4.7%) than in the TrA group.

The differences in our MTX group's results may be due to several factors. Firstly, we administered MTX injections to patients three times at 4‐week intervals, whereas the two mentioned studies provided four MTX injections at 3‐week intervals. Second, the possibility of a higher number of patients experiencing disease relapse or being treatment‐resistant in the MTX group, and third, the larger proportion of male patients in the MTX group and the higher prevalence of androgenetic alopecia among males [25]. The point that alopecia itself is one of the side effects of using MTX should also be taken into consideration [10].

A literature review conducted by Browne et al. determined that there is extremely poor evidence on the effectiveness of MTX in either promoting regrowth of alopecia totalis or in maintaining it after spontaneous or drug‐induced regrowth occurs [26]. A systematic review and meta‐analysis about MTX use for AA revealed that when MTX is used in combination with corticosteroids, response rates are greater than when MTX is used alone for AA. Additionally, a significant number of recurrences during the tapering phase of therapy were reported. The paper also highlights the lack of prospective controlled trials and long‐term data, indicating a need for more rigorous studies to better understand the efficacy and safety of MTX in treating AA [15].

Similar to other studies demonstrating improvement with intralesional TrA treatment in AA patients [8], we observed an average improvement of 54.36% in patients' SALT score percentage. We found that 80% of patients treated with 5–10 mg of TrA monthly for 3 months experienced 50% or more hair regrowth. A systematic review and meta‐analysis on the efficacy of various concentrations of intralesional TrA for AA reported pooled hair regrowth rates of 62.3% (CI: 50.6%–72.8%, *p* = 0.04) with concentrations under 5 mg/mL, 80.9% (CI: 71%–88%, *p* < 0.005) with 5 mg/mL, and 76.4% (CI: 67.1%–83.7%, *p* < 0.005) with 10 mg/mL concentrations [27].

Our study found a general downward trend in trichoscopic findings (yellow dots, black dots, empty follicles, absent follicular opening) in both treatment groups over time; however, only the diminution of black dots in the MTX group was statistically significant. Black dots are connected with disease activity in AA, according to an article about trichoscopy results and disease activity [28] This suggests that while MTX did not demonstrate clinical improvement in our patients, it did have some impact on reducing disease activity. The reduction of black and yellow dots in the TrA group of our study aligns with previous research [29, 30], but their studies showed a more dramatic and significant decrease compared to ours.

In the first published study comparing the effects of intralesional MTX versus TrA for treating localized AA, adverse effects were minor and transient, including hypo/hyper pigmentation, erythema, erosions, and atrophy, with a significantly higher prevalence in the MTX group [17]. In our RCT, both treatments were generally safe and well‐tolerated, with minimal adverse effects reported. Erythema was observed in 15% of patients in both groups, and hypopigmentation was noted in one patient in the MTX group. No laboratory abnormalities were detected in either group.

Patient satisfaction is a crucial component of treatment success, particularly in chronic conditions like AA, where quality of life can be significantly impacted by the disease [31]. Contrary to the findings of Hamdino et al. [17], our study observed higher patient satisfaction in the TrA group, as patients in this group had demonstrated a more favorable response to the treatment.

Similar to the findings of Ganjoo et al. [30], we did not observe any correlation between age, sex, and treatment response in any of the groups. However, unlike their results and those of Muhaidat JM [2] we did not identify any association between the duration of the disease and treatment response in any of the groups.

The present study's findings have important implications for clinical practice. Due to greater efficacy and patient satisfaction with intralesional TrA, it should continue to be a first‐line treatment for limited patchy AA. Our results show that intralesional MTX is not consistently effective as monotherapy, although it may still be useful in certain patients or in combination therapies. This is important because some earlier research has shown promising results with intralesional MTX, which could lead physicians to prescribe it without second thoughts. Our different results point out the importance of caution in introducing new therapies and the need to closely observe the treatment responses.

### 4.1 Limitations

There are some limitations to this study that need to be considered. First, the relatively small sample size (20 patients per group) reduces the statistical power and the generalizability of our findings; larger multicenter studies are needed. Second, the lack of a placebo control group limits our capacity to separate treatment effects from the natural course of AA. Third, the wide heterogeneity in response seen in the MTX group, evidenced by the large SD of SALT score improvement, suggests that MTX has a mixed response profile. Future studies should explore patient‐specific factors that may influence response to treatment. Also, the 6‐month period of follow‐up may be too short to fully evaluate long‐term efficacy and relapse rates because of the chronic and relapsing nature of AA. Longer‐term studies are required to assess the durability of treatment outcomes. Another limitation is the risk of performance bias, since the treating physician was not blinded to the treatment. Nevertheless, all injections were carried out by a very experienced dermatology professor according to a standardized injection protocol, so this risk could be limited. However, it is impossible to completely rule out an unconscious influence on injection technique. Although a blinded investigator conducted the outcome assessment, we did not formally evaluate the effectiveness of blinding, which could have limited the internal validity of our findings. At last, while we noted some significant trichoscopic findings, such as persistent white dots in the MTX group and exclamation mark hairs in the TrA group, our study was not designed to investigate their possible diagnostic or prognostic value. The clinical significance of these findings needs to be evaluated in future studies.

## 5 Conclusions

Our results highlight the potential efficacy of intralesional TrA in managing AA, while suggesting that the efficacy of intralesional MTX monotherapy may be inconsistent and less reliable as a treatment option for AA. Additional research is needed to assess the true efficacy of intralesional MTX in larger patient populations with extended follow‐up periods, as well as to determine the most effective treatment regimen for this medication.

## Author Contributions

Study conception and design: Narges Ghandi and Robabeh Abedini. Gathering, analysis, and interpretation of results: Atiyeh Rashisi and Nasim Tootoonchi. Draft manuscript preparation: Hanie Babaie and Fatemeh Saberi. Manuscript edit and revision: Narges Ghandi, Fatemeh Saberi, and Hanie Babaie. All authors reviewed the results and approved the final version of the manuscript.

## Conflicts of Interest

The authors declare no conflicts of interest.

## Data Availability

The data that support the findings of this study are available on request from the corresponding author. The data are not publicly available due to privacy or ethical restrictions.

## References

[1] D. Dahabreh , S. Jung , Y. Renert‐Yuval , J. Bar , E. del Duca , and E. Guttman‐Yassky , “Alopecia Areata: Current Treatments and New Directions,” American Journal of Clinical Dermatology 24, no. 6 (2023): 895–912. 37606849 10.1007/s40257-023-00808-1

[2] J. M. Muhaidat , F. Al‐Qarqaz , Y. Khader , D. M. Alshiyab , H. Alkofahi , and M. Almalekh , “A Retrospective Comparative Study of Two Concentrations of Intralesional Triamcinolone Acetonide in the Treatment of Patchy Alopecia Areata on the Scalp,” Clinical, Cosmetic and Investigational Dermatology 13 (2020): 795–803. 33173320 10.2147/CCID.S280855 PMC7646378

[3] I. Šutić Udović , N. Hlača , L. P. Massari , I. Brajac , M. Kaštelan , and M. Vičić , “Deciphering the Complex Immunopathogenesis of Alopecia Areata,” International Journal of Molecular Sciences 25, no. 11 (2024): 5652. 38891839 10.3390/ijms25115652 PMC11172390

[4] A. Waśkiel‐Burnat , M. Osińska , A. Salińska , et al., “The Role of Serum Th1, Th2, and Th17 Cytokines in Patients With Alopecia Areata: Clinical Implications,” Cells 10, no. 12 (2021): 3397. 34943905 10.3390/cells10123397 PMC8699846

[5] H. A. Blair , “Ritlecitinib: First Approval,” Drugs 83, no. 14 (2023): 1315–1321. 37556041 10.1007/s40265-023-01928-y PMC10556173

[6] “Barcitinib,” (2024).

[7] K. Chanprapaph , C. Pomsoong , C. Kositkuljorn , and P. Suchonwanit , “Intramuscular Corticosteroid Therapy in the Treatment of Alopecia Areata: A Time‐To‐Event Analysis,” Drug Design, Development and Therapy 16 (2022): 107–116. 35027820 10.2147/DDDT.S342179 PMC8752075

[8] M. Kumaresan , “Intralesional Steroids for Alopecia Areata,” International Journal of Trichology 2, no. 1 (2010): 63–65. 21188031 10.4103/0974-7753.66920 PMC3002419

[9] K. Lepe , H. A. Syed , and P. M. Zito , Alopecia Areata (Treasure Island, 2024). 30725685

[10] M. Hanoodi and M. Mittal , Methotrexate (StatPearls, 2024). 32310574

[11] Y. Tanaka , “Subcutaneous Injection of Methotrexate: Advantages in the Treatment of Rheumatoid Arthritis,” Modern Rheumatology 33, no. 4 (2023): 633–639. 36525530 10.1093/mr/roac156

[12] L. L. Westerkam , D. B. McShane , E. L. Nieman , and D. S. Morrell , “Treatment Options for Alopecia Areata in Children and Adolescents,” Paediatric Drugs 26, no. 3 (2024): 245–257. 38466519 10.1007/s40272-024-00620-2

[13] A. Asilian , F. Fatemi , Z. Ganjei , A. H. Siadat , F. Mohaghegh , and M. Siavash , “Oral Pulse Betamethasone, Methotrexate, and Combination Therapy to Treat Severe Alopecia Areata: A Randomized, Double‐Blind, Placebo‐Controlled, Clinical Trial,” Iranian Journal of Pharmaceutical Research 20, no. 1 (2021): 267–273. 34400956 10.22037/ijpr.2020.113868.14536 PMC8170764

[14] H. Albela , S. Begum , A. L. Wee , N. Ponnuthurai , and K. F. Leong , “Efficacy and Tolerability of Methotrexate in the Treatment of Severe Paediatric Alopecia Areata,” Skin Appendage Disorders 8, no. 3 (2022): 206–210. 35707294 10.1159/000521238 PMC9149515

[15] K. Phan , V. Ramachandran , and D. F. Sebaratnam , “Methotrexate for Alopecia Areata: A Systematic Review and Meta‐Analysis,” Journal of the American Academy of Dermatology 80, no. 1 (2019): 120–127.e2. 30003990 10.1016/j.jaad.2018.06.064

[16] M. Kinoshita‐Ise , M. Sachdeva , S. A. Martinez‐Cabriales , N. H. Shear , and P. Lansang , “Oral Methotrexate Monotherapy for Severe Alopecia Areata: A Single Center Retrospective Case Series,” Journal of Cutaneous Medicine and Surgery 25, no. 5 (2021): 490–497. 33715460 10.1177/1203475421995712

[17] M. Hamdino , R. A. El‐Barbary , and H. M. Darwish , “Intralesional Methotrexate Versus Triamcinolone Acetonide for Localized Alopecia Areata Treatment: A Randomized Clinical Trial,” Journal of Cosmetic Dermatology 21, no. 2 (2022): 707–715. 33749975 10.1111/jocd.14090

[18] M. L. Elsaie and M. S. Hasan , “Successful Treatment of Long‐Standing Alopecia Totalis With Intralesional Methotrexate,” Journal of Cosmetic Dermatology 21, no. 2 (2022): 855–856. 33780590 10.1111/jocd.14117

[19] A. Rodríguez‐Villa Lario , Á. Aguado‐García , J. J. Andrés‐Lencina , et al., “Successful Response to a Combination of Intralesional Methotrexate and Fractional CO(2) Laser in Refractory Alopecia Areata: Case Report,” Skin Appendage Disorders 8, no. 6 (2022): 486–491. 36407646 10.1159/000524672 PMC9672868

[20] A. Lyakhovitsky , A. Aronovich , S. Gilboa , S. Baum , and A. Barzilai , “Alopecia Areata: A Long‐Term Follow‐Up Study of 104 Patients,” Journal of the European Academy of Dermatology and Venereology 33, no. 8 (2019): 1602–1609. 30887594 10.1111/jdv.15582

[21] E. T. Landis and R. O. Pichardo‐Geisinger , “Methotrexate for the Treatment of Pediatric Alopecia Areata,” Journal of Dermatological Treatment 29, no. 2 (2018): 145–148. 28627278 10.1080/09546634.2017.1341608

[22] C. A. Bangert and M. I. Costner , “Methotrexate in Dermatology,” Dermatologic Therapy 20, no. 4 (2007): 216–228. 17970887 10.1111/j.1529-8019.2007.00135.x

[23] R. M. Farag , S. A. Salem , A. A. Afify , and W. A. E. Ahmed , “Intralesional Methotrexate for Treatment of Alopecia Areata: Clinical Evaluation and Effect on Lesional TNF Alpha,” QJM: An International Journal of Medicine 116, no. Supplement_1 (2023): hcad069‐239.

[24] S. Rafique , T. Hassan , M. K. Shahzad , N. Aliya , S. Rafique , and S. Rafique , “Comparison of Intra‐Lesional Methotrexate and Intra‐Lesional Triamcinolone Acteonid in the Treatment of Localized Alopecia Areata,” Pakistan Journal of Medical and Health Sciences 17 (2023): 179–181.

[25] N. Otberg , A. M. Finner , and J. Shapiro , “Androgenetic Alopecia,” Endocrinology and Metabolism Clinics of North America 36, no. 2 (2007): 379–398. 17543725 10.1016/j.ecl.2007.03.004

[26] R. Browne , L. Stewart , and H. C. Williams , “Is Methotrexate an Effective and Safe Treatment for Maintaining Hair Regrowth in People With Alopecia Totalis? A Critically Appraised Topic,” British Journal of Dermatology 179, no. 3 (2018): 609–614. 29777625 10.1111/bjd.16796

[27] B. E. Yee , Y. Tong , A. Goldenberg , and T. Hata , “Efficacy of Different Concentrations of Intralesional Triamcinolone Acetonide for Alopecia Areata: A Systematic Review and Meta‐Analysis,” Journal of the American Academy of Dermatology 82, no. 4 (2020): 1018–1021. 31843657 10.1016/j.jaad.2019.11.066

[28] B. M. Vyshak , B. R. Doshi , and B. S. Manjunathswamy , “1‐Year Hospital‐Based Observational Study of Trichoscopy Findings and Disease Activity in Alopecia Areata,” Indian Dermatology Online Journal 11, no. 6 (2020): 965–969. 33344348 10.4103/idoj.IDOJ_19_20 PMC7735008

[29] S. Srivastava , S. Goyal , K. S. Dhillon , and N. Singh , “Dermoscopic Evaluation of Therapeutic Response to Intralesional Triamcinolone Acetonide in the Treatment of Alopecia Areata,” International Journal of Advances in Medicine 4 (2017): 1175–1183.

[30] S. Ganjoo and D. M. Thappa , “Dermoscopic Evaluation of Therapeutic Response to an Intralesional Corticosteroid in the Treatment of Alopecia Areata,” Indian Journal of Dermatology, Venereology and Leprology 79, no. 3 (2013): 408–417. 23619446 10.4103/0378-6323.110767

[31] A. Toussi , V. R. Barton , S. T. le , O. N. Agbai , and M. Kiuru , “Psychosocial and Psychiatric Comorbidities and Health‐Related Quality of Life in Alopecia Areata: A Systematic Review,” Journal of the American Academy of Dermatology 85, no. 1 (2021): 162–175. 32561373 10.1016/j.jaad.2020.06.047 PMC8260215

